# Validity, Responsiveness, Long-Term Stability, and Minimal Detectable Change in the Six-Minute Walk Test in Individuals with Schizophrenia Receiving Exercise Interventions

**DOI:** 10.3390/healthcare14172885

**Published:** 2026-09-07

**Authors:** Chyi-Rong Chen, Tzu-Ting Chen, Yu-Chi Huang, Pao-Yen Lin, Liang-Jen Wang, Tien-Ni Wang, Mei-Hsiang Chen, Keh-chung Lin

**Affiliations:** 1 Department of Psychiatry, Kaohsiung Chang Gung Memorial Hospital and Chang Gung University College of Medicine, Kaohsiung 833401, Taiwan; ccr776@cgmh.org.tw (C.-R.C.); tracy5824@cgmh.org.tw (T.-T.C.); yuchihuang@cgmh.org.tw (Y.-C.H.); py1029@cgmh.org.tw (P.-Y.L.); 2 School of Occupational Therapy, College of Medicine, National Taiwan University, Taipei 100025, Taiwan; tnwang@ntu.edu.tw; 3 Department of Child and Adolescent Psychiatry, Kaohsiung Chang Gung Memorial Hospital and Chang Gung University College of Medicine, Kaohsiung 833401, Taiwan; anus78@cgmh.org.tw; 4 Division of Occupational Therapy, Department of Physical Medicine and Rehabilitation, National Taiwan University Hospital, Taipei 100229, Taiwan; 5 Department of Occupational Therapy, College of Medical Science and Technology, Chung Shan Medical University, Taichung 402306, Taiwan; cmh@csmu.edu.tw; 6 Occupational Therapy Room, Chung Shan Medical University Hospital, Taichung 402306, Taiwan

**Keywords:** schizophrenia, mind–body therapies, six-minute walk test, psychometrics, physical fitness, dual-task test

## Abstract

**Highlights:**

**What are the main findings?**

- The 6-Minute Walk Test (6MWT) demonstrated excellent long-term stability of scores (ICC = 0.96; MDC_95_ = 37.19 m) and high responsiveness (SRM = 1.08).
- Moderate correlations of the 6MWT with Timed Up-and-Go and 30-Second Chair Stand Tests supported its construct validity as a sensitive measure of functional exercise capacity in schizophrenia.

**What is the implication of the main finding?**

- The 6MWT can serve as a practical and sensitive outcome measure for monitoring physical function in psychiatric rehabilitation.

**Abstract:**

**Background**: Reduced physical fitness in individuals with schizophrenia contributes to disability and health disparities, highlighting the need for valid and responsive field measures in exercise interventions. **Objective**: This study examined the long-term stability, construct validity, and responsiveness of the Six-Minute Walk Test (6MWT) in individuals with schizophrenia undergoing structured exercise interventions. **Methods**: This secondary analysis included 70 participants from two randomized controlled trials of 12-week Baduanjin or brisk walking interventions. The cohorts were pooled after confirming comparable baseline characteristics and no significant group-by-time interaction in 6MWT change. Baseline and post-intervention assessments included the 6MWT, the Timed Up-and-Go (TUG), the cognitive dual-task TUG (TUGcognitive), and the 30-Second Chair Stand Test (30CST). Long-term stability of 6MWT scores was assessed using the intraclass correlation coefficient (ICC), and measurement error was estimated using the standard error of measurement (SEM) and minimal detectable change at the 95% confidence level (MDC_95_). Construct validity was examined using Spearman correlations (ρ), and responsiveness was examined using the standardized response mean (SRM) and Cohen’s *d*. **Results**: The 6MWT showed excellent long-term stability (ICC = 0.96, 95% CI: 0.93–0.97), with SEM = 13.41 m and MDC_95_ = 37.19 m. Construct validity was supported by correlations with the TUG (ρ = −0.54, *p* < 0.001), 30CST (ρ = 0.45, *p* < 0.001), and TUGcognitive (ρ = −0.36, *p* = 0.002). Responsiveness was large (SRM = 1.08, 95% CI: 0.84–1.31), with a complementary effect size of *d* = 0.32 (95% CI = 0.24 to 0.42), and 24.29% of participants exceeded the MDC_95_ threshold. **Conclusions**: The 6MWT demonstrated appropriate long-term stability, construct validity, and responsiveness as a measure of functional exercise capacity in schizophrenia, supporting its use for evaluating exercise outcomes in psychiatric rehabilitation. Because the present psychometric analysis did not include a non-exercise comparison group, the responsiveness estimates reflect within-intervention change and should not be interpreted as causal treatment effects of exercise. Larger studies are needed to identify responder characteristics and trajectories of changes in test performance.

## 1. Introduction

Individuals with schizophrenia have markedly elevated cardiometabolic risk, largely related to sedentary behavior [1], which contributes to excess cardiovascular mortality and reduced life expectancy [2,3]. These findings support the integration of structured aerobic and mind–body exercise into psychiatric rehabilitation to improve cardiometabolic health, physical fitness, and functional recovery [4,5]. Accumulating evidence indicates that structured exercise interventions, including aerobic and mind–body modalities, improve psychopathology, cognitive function, and cardiometabolic health in individuals with schizophrenia [5,6,7]. However, these benefits may diminish without continued maintenance [8]. Baduanjin and brisk walking represent practical, low-cost interventions that target both physical and cognitive domains. Baduanjin integrates diaphragmatic breathing with coordinated limb movements to enhance postural control and attentional focus, whereas brisk walking is widely acceptable, scalable, and easily implemented. Both are light-to-moderate-intensity exercise modalities [9,10]. Their inclusion in psychiatric rehabilitation highlights the need for valid, portable outcome measures suitable for mental health settings.

The six-minute walk test (6MWT) is a standardized submaximal aerobic measure valued for its simplicity, safety, and clinical utility [11]. Requiring only a stopwatch and a marked corridor, it offers a low-cost, easily administered alternative to laboratory-based cardiopulmonary testing [11]. The test evaluates the integrated responses of the cardiovascular, pulmonary, circulatory, and neuromuscular systems under submaximal exertion, making it particularly suitable for individuals who are unable to complete maximal exercise testing [12]. Clinically, the 6MWT is practical, requires minimal equipment, and can be administered without specialized training, supporting its use in individuals with schizophrenia. Because most daily activities are performed at submaximal intensity, the test provides an ecologically valid measure of real-world functional capacity. Its self-paced and well-tolerated nature also suggests its potential as a reliable, safe, and responsive outcome for evaluating exercise-related functional change, although further validation is warranted.

The 6MWT has emerged as a practical submaximal measure of functional exercise capacity, yet evidence regarding its psychometric properties in severe mental illness remains limited. In schizophrenia, reference data from 237 Taiwanese adults identified age and height as key predictors of walking distance and provided sex- and age-stratified norms to support clinical interpretation [13]. Notably, this Taiwanese reference study used a 25-m walkway with testing procedures based on the American Thoracic Society recommendations [13]. A 25-m walkway has also been used in previous schizophrenia-specific studies demonstrating high test–retest reliability and establishing MDC values for the 6MWT [12,14]. A systematic review of 16 studies found that individuals with schizophrenia generally walked shorter distances than healthy controls and showed high test–retest reliability, although evidence for validity and responsiveness remained limited and methodologically heterogeneous [15]. Prior research also reported excellent reliability (ICC = 0.94–0.96) and MDC values of 50 to 56 m, supporting the reproducibility of the 6MWT in this population [14]. In addition, criterion validity has been supported by moderate-to-strong correlations with peak oxygen uptake in men with schizophrenia, whereas previous research has also reported associations between 6MWT performance and clinical characteristics such as body mass index, medication dosage, and symptom severity [12].

However, evidence regarding responsiveness remains limited. Earlier intervention studies seldom reported significant improvements in 6MWT distance, likely because of small sample sizes and heterogeneous protocols. A study using the 6MWT in individuals with serious mental illness did not report standardized responsiveness indices such as effect sizes or SRMs [16]. Moreover, evidence suggests that aerobic exercise interventions lasting at least 12 weeks may be associated with improvements in global cognition in individuals with schizophrenia [17]. Nevertheless, multimodal exercise interventions may improve physical and psychological outcomes without corresponding gains in 6MWT performance, underscoring the need to clarify the responsiveness of the 6MWT to specific exercise modalities and intervention durations in schizophrenia. Because short-interval retest data were unavailable, the present study did not evaluate conventional test–retest reliability. Instead, the ICC was used to examine the long-term stability of 6MWT scores across baseline and 12-week post-intervention assessments. Therefore, a schizophrenia-specific psychometric study examining the construct validity, responsiveness, long-term stability of the scores, and measurement error of the 6MWT in a well-characterized rehabilitation cohort is needed to address these gaps and inform clinical practice and future trials. We hypothesized that the 6MWT would demonstrate acceptable construct validity, defined as correlations with an absolute magnitude of ρ ≥ 0.30 in the hypothesized directions; large responsiveness, defined as an SRM ≥ 0.80; and excellent long-term stability, defined as an ICC > 0.80, following 12 weeks of Baduanjin or brisk walking interventions in individuals with schizophrenia.

## 2. Materials and Methods

### 2.1. Study Design and Participants

**Figure 1:**
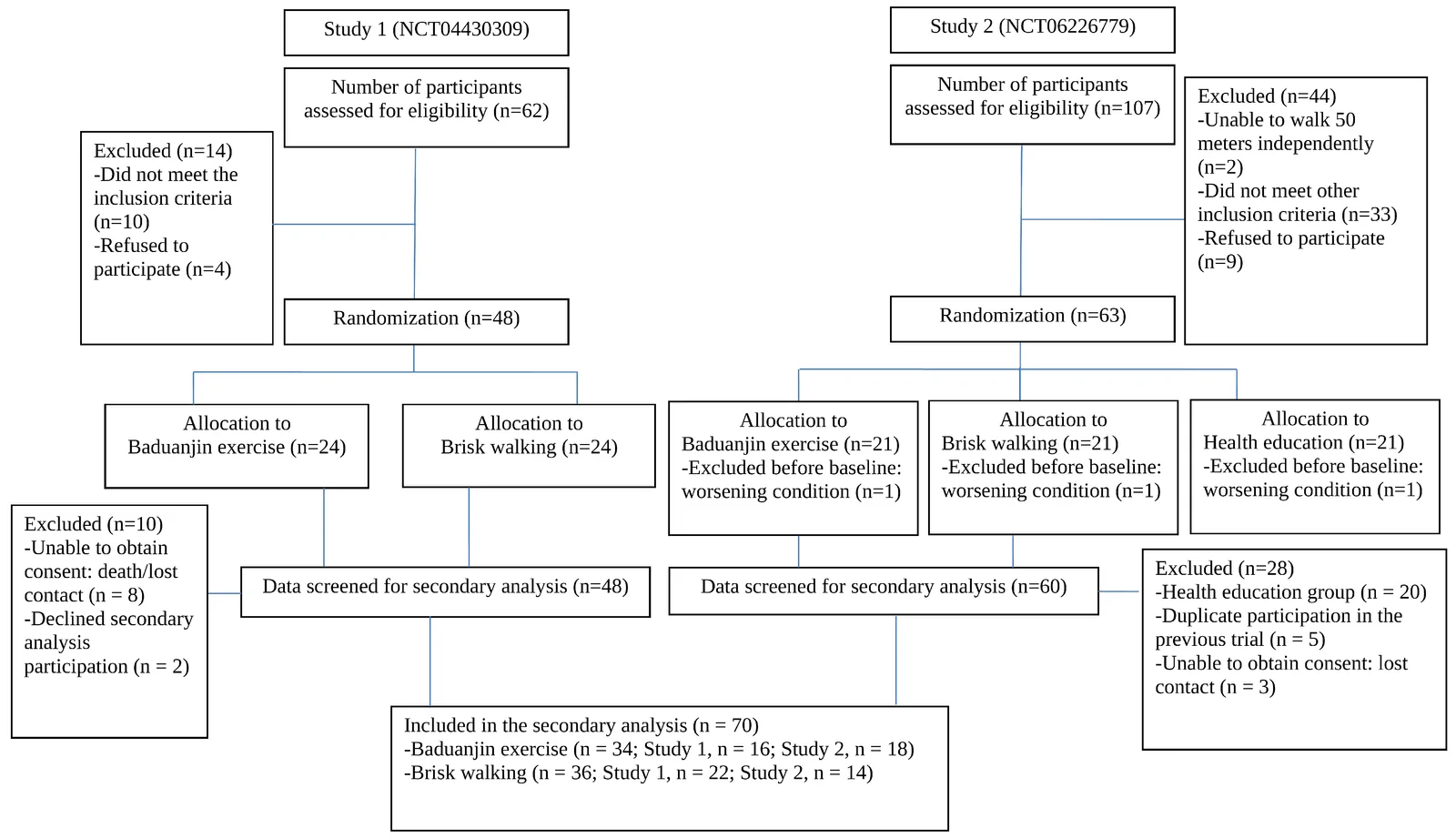
Flow diagram of participant selection for the secondary analysis.

This study was based on a secondary analysis of data obtained from 70 participants who participated in two randomized controlled trials [10,18] (identifiers: NCT04430309 and NCT06226779). A total of 108 participants from the two source trials were screened for the present secondary analysis. Of the 48 participants studied by Chen et al. [10], 10 were excluded, resulting in 38 participants. Of the 60 participants studied by Chen et al. [18], 28 were excluded, resulting in 32 participants. Thus, the final analytic sample included 70 participants, as detailed in Figure 1. One of the parent trials [18] also included a health education arm. The present secondary analysis was restricted to participants receiving structured exercise interventions because its objective was to evaluate the psychometric properties of the 6MWT in the context of exercise rehabilitation rather than to estimate the causal efficacy of exercise compared with a non-exercise condition. Accordingly, participants in the health education arm were not included in the present psychometric analytic sample.

Data from the Baduanjin and brisk walking groups were pooled because both were structured 12-week exercise interventions, used the same 6MWT assessment procedures, and were designed to improve physical function in individuals with schizophrenia. The two cohorts showed no significant differences in baseline characteristics. In addition, no significant group by time interaction was found for the 6MWT, supporting the pooling of data from the two trials for the present analyses. Participants included in the present analysis had been randomized to either the Baduanjin or brisk walking group in the parent trials. Only participants with complete data available for all functional assessments in the analytic dataset were included in the analysis. For individuals who participated in more than one clinical trial, only data from their first trial were included to minimize the risk of data contamination. Participants were eligible for inclusion if they met the following criteria: (1) a DSM-5 diagnosis of schizophrenia [19]; (2) age of 20 years or older; (3) clinical stability with no medication adjustments during the preceding month. Independent ambulation of at least 50 m was an eligibility criterion in Chen et al. [18], whereas this criterion was not specified in Chen et al. [10]. In Chen et al. [18], this requirement served as a pragmatic safety and feasibility criterion to ensure that participants could complete the walking-based assessments without physical assistance. Exclusion criteria were: (1) severe physical conditions such as cardiovascular or musculoskeletal disorders; (2) visual or auditory impairments likely to affect test performance; (3) requirement for hospitalization; or (4) severe withdrawal manifestations or profound intellectual disability. The studies were reviewed and approved by the Institutional Review Board of Kaohsiung Chang Gung Memorial Hospital. All participants provided written informed consent to participate in this study. Because this was a secondary analysis of two completed randomized controlled trials, the sample size was determined by the number of eligible participants included in the analytic dataset. No a priori sample-size calculation was performed for the SRM because an anticipated SRM was not prespecified.

### 2.2. Intervention and Procedures

Participants took part in the Baduanjin or brisk walking intervention. The Baduanjin program consisted of eight consecutive movements, each emphasizing specific body regions: (1) Two Hands Hold Up the Heavens; (2) Drawing the Bow to Shoot the Eagle; (3) Separate Heaven and Earth; (4) Wise Owl Gazes Backward; (5) Sway the Head and Shake the Tail; (6) Two Hands Hold the Feet to Strengthen the Kidneys and Waist; (7) Clench the Fists and Glare Fiercely; and (8) Bouncing on the Toes. All sessions were led by a certified occupational therapist in small groups of six to eight participants, lasting for 60 min per session and conducted two to three times per week for 12 weeks.

Participants in the brisk walking group engaged in 60-min sessions held two to three times per week over a 12-week period, while continuing their routine medical and psychosocial treatments. Each session included a 10-min warm-up involving range-of-motion exercises, 40 min of brisk walking, and a 10-min cool-down with stretching. Sessions were conducted in groups of six to eight participants. The exercise intensity was maintained at a light to moderate level.

Within each parent trial, baseline and post-intervention assessments were administered by the same rater. Different well-trained research assistants served as raters in the two parent trials, both of whom were blinded to group allocation and uninvolved in participant screening or intervention delivery. All assessments were performed in accordance with the standardized sequence outlined in the study protocols of the respective clinical trials.

### 2.3. Measures

#### 2.3.1. The Six-Minute Walk Test (6MWT)

The 6MWT is a practical, submaximal measure of functional exercise capacity that can be readily administered in clinical and rehabilitation settings with minimal equipment. The 6MWT was conducted along a standardized 25-m indoor corridor, which was the longest suitable walkway available at the study site. Participants were instructed to walk back and forth for six minutes and received standardized verbal encouragement at fixed intervals in accordance with the American Thoracic Society guidelines [11]. The total distance covered during this period was recorded to the nearest meter, with greater distances reflecting superior aerobic endurance and cardiovascular fitness.

#### 2.3.2. The Timed Up and Go Test (TUG)

The Timed Up and Go Test (TUG) is a standardized measure of functional mobility, balance, and agility. Participants were instructed to rise from a standard chair, walk 3 m at a comfortable and safe pace, turn around a marker, return to the chair, and sit down. Completion time was recorded in seconds using a stopwatch, with shorter times indicating better mobility and postural control [20]. The TUG has been shown to be a reliable and valid assessment in older adults and clinical populations, including individuals with psychiatric conditions [20].

#### 2.3.3. Dual-Task Performance

Dual-task performance was assessed using the cognitive Timed Up and Go Test (TUGcognitive), which measures mobility while performing a concurrent mental task. Participants were instructed to complete the TUG as quickly and safely as possible while counting backward by threes from a randomly selected number between 80 and 99 [21]. No explicit priority was assigned to either gait or cognitive performance, and cognitive accuracy was not recorded. The total completion time was recorded with a stopwatch [21]. One practice trial and three official trials were administered, and the mean time of the three official trials was used for analysis.

#### 2.3.4. The 30-Second Chair Stand Test (30CST)

The 30-Second Chair Stand Test (30CST) is a measure of lower-limb strength and muscular endurance suitable for clinical and community settings [22]. Participants sat upright in a standard chair placed against a wall, with their arms folded across the chest. They were instructed to stand up fully and sit down as many times as possible within 30 s [22]. The number of completed repetitions was recorded, with higher scores indicating better lower extremity strength and endurance. The 30CST has been validated as an indicator of mobility and fall risk in various populations [22].

### 2.4. Data Analysis

Statistical analyses were conducted using SPSS version 25.0 (IBM Corp., Armonk, NY, USA). Data are presented as means and standard deviations. Baseline comparability between the Baduanjin and brisk walking cohorts was examined using independent-sample *t* tests for continuous variables and the chi-square test or Fisher’s exact test, as appropriate, for categorical variables. These baseline comparisons were conducted for descriptive purposes; therefore, no adjustment for multiple comparisons was applied, and the resulting *p*-values were interpreted descriptively rather than as confirmatory hypothesis tests. To examine whether pooling the two exercise modalities was appropriate, an exploratory 2 × 2 repeated-measures analysis of variance was conducted for 6MWT distance, with time (baseline and post-intervention) as the within-subject factor and intervention group (Baduanjin and brisk walking) as the between-subject factor. The group-by-time interaction was used to determine whether the magnitude of change differed between exercise modalities. Paired *t*-tests were used to examine pre- to post-intervention changes in walking distance, functional mobility, lower extremity strength, and dual-task performance. To account for multiple comparisons, the significance level was adjusted to 0.0125 (0.05/4) using the Bonferroni correction [23].

Construct validity was examined using Spearman’s rho correlations between baseline 6MWT scores and baseline TUG, TUG-cognitive, and 30CST scores. Correlations with an absolute magnitude of *ρ* ≥ 0.30 in the hypothesized direction were considered to support construct validity. Statistical significance was set at *p* < 0.05. Long-term stability of the 6MWT scores was evaluated using the intraclass correlation coefficient (ICC) with 95% confidence intervals (95% CIs), based on a two-way mixed-effects model with absolute agreement. The ICC was estimated using baseline and post-intervention data from 70 participants with schizophrenia who completed a 12-week Baduanjin or brisk walking intervention. ICC values > 0.80 were considered to indicate excellent long-term stability of the scores [24]. The standard error of measurement (SEM) and minimal detectable change at the 95% confidence level (MDC_95_) were also calculated. For the responder analysis, participants were classified as responders if the increase in 6MWT distance from baseline to post-intervention exceeded the calculated MDC_95_. Thus, the responder criterion was defined one-directionally to represent improvement in 6MWT performance.

Responsiveness was assessed using the standardized response mean (SRM) and its 95% CIs. The SRM was calculated as the mean change divided by the standard deviation of the change scores, representing the sensitivity of the 6MWT to detect change over time. SRM values of 0.20–0.49, 0.50–0.79, and ≥0.80 were interpreted as small, moderate, and large responsiveness, respectively [25]. Effect size estimates were calculated using Cohen’s *d*, with values of 0.20, 0.50, and 0.80 indicating small, medium, and large effects, respectively [26]. Cohen’s *d* was calculated as d_p and standardized by the pooled standard deviation of the pre- and post-intervention scores, i.e., √[(SD^2^pre + SD^2^post)/2]. Given the high correlation between pre- and post-intervention scores (Pearson’s *r* = 0.957), its 95% confidence interval was estimated using the MAG method, which maintains valid coverage under high-correlation conditions [27].

## 3. Results

### 3.1. Participant Characteristics

A total of 70 participants were included in the analysis (37 men and 33 women), with a mean age of 49.21 ± 9.92 years and a mean body mass index (BMI) of 26.05 ± 4.72 kg/m^2^. The average duration of illness was 26.35 ± 8.48 years, and the mean daily chlorpromazine-equivalent dosage was 448.41 ± 236.90 mg/day. The mean Clinical Global Impression–Severity (CGI-S) score was 3.40 ± 0.67, reflecting mild-to-moderate symptom severity (Table 1). Baseline demographic, clinical, and functional characteristics were descriptively similar between the Baduanjin and brisk walking cohorts, with no statistically significant differences observed (all *p* > 0.05). The paired *t*-tests revealed significant differences between pretest and posttreatment scores for the 6MWT (*p* < 0.001), the TUG (*p* < 0.001), the 30CST (*p* < 0.001), and the TUG-cognitive (*p* < 0.001), indicating improvements in walking endurance, functional mobility, lower-limb strength, and dual-task performance following the interventions.

### 3.2. Long-Term Stability and Measurement Error

**Figure 2:**
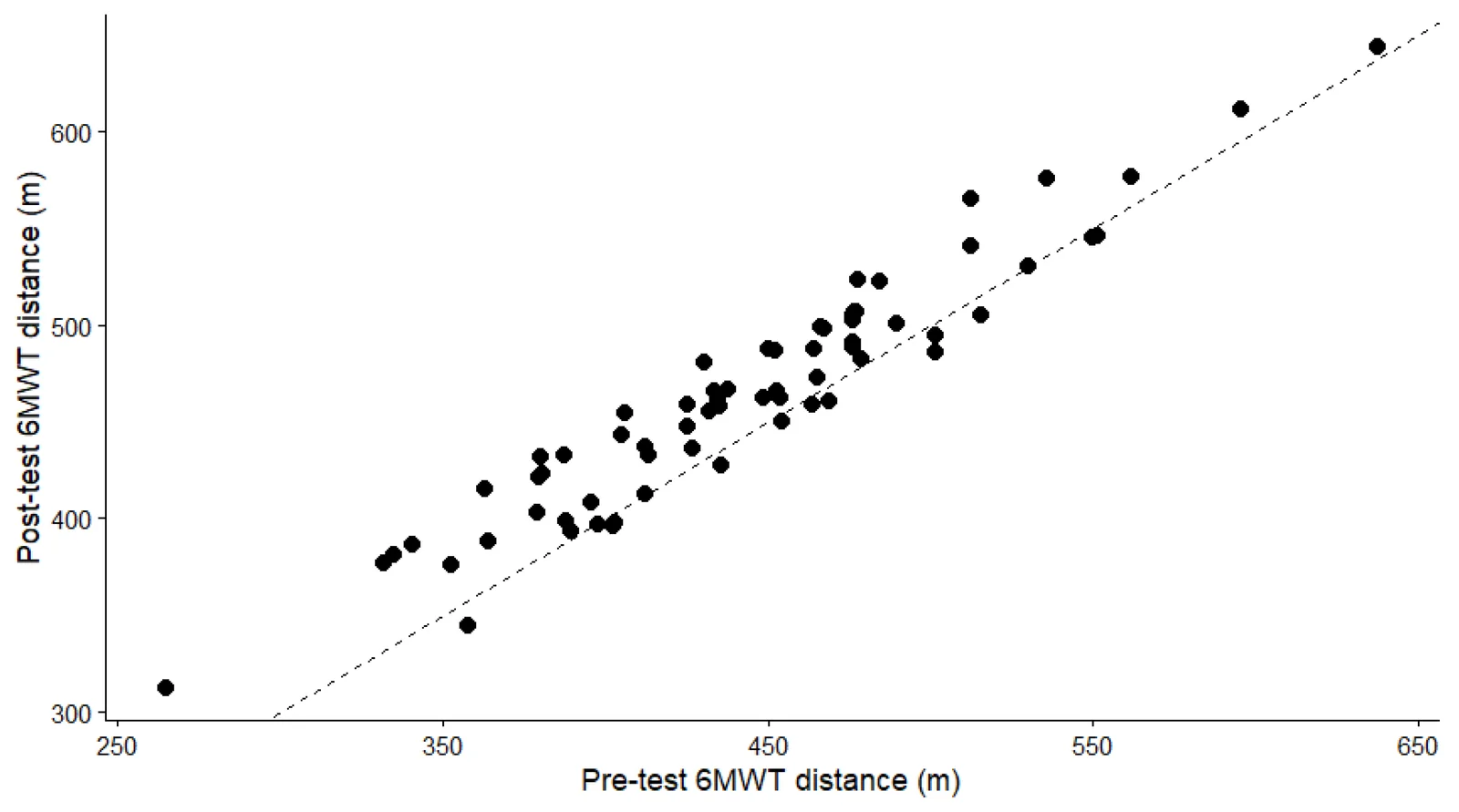
Scatterplot of baseline and post-intervention 6-min walk test distances. Each point represents performance of one participant. The dashed diagonal line represents the line of identity (y=x), indicating equal baseline and post-intervention scores.

The 6MWT demonstrated excellent stability across the 12-week intervention period, with an intraclass correlation coefficient (ICC) of 0.96 (95% CI: 0.93–0.97, *p* < 0.001). The standard error of measurement (SEM) was 13.41 m, and the minimal detectable change at the 95% confidence level (MDC_95_) was 37.19 m. These findings indicate a high degree of stability in 6MWT scores across the 12-week assessment interval. Figure 2 illustrates the association between baseline and post-intervention 6MWT distances. Of the 70 participants, 55 (78.57%) were above the line of identity, 13 (18.57%) were below the line, and 2 (2.86%) were on the line, indicating that most participants showed improvement in 6MWT performance after the intervention.

### 3.3. Construct Validity

Construct validity was evaluated using Spearman’s rho correlations between baseline 6MWT performance and other physical function measures. The 6MWT showed a moderate negative correlation with the TUG (ρ = −0.54, *p* < 0.001), indicating that participants with better functional mobility walked longer distances. A moderate positive correlation was observed with the 30CST (ρ = 0.45, *p* < 0.001), suggesting that greater lower-limb strength was associated with longer walking distance. The TUG-cognitive showed a moderate negative correlation with the 6MWT (ρ = −0.36, *p* = 0.002), indicating that dual-task interference was negatively associated with walking performance. Together, these findings support the construct validity of the 6MWT as a multidimensional indicator of functional mobility and lower-limb endurance.

### 3.4. Responsiveness

The exploratory group-by-time analysis showed no significant interaction effect for 6MWT distance, indicating that the magnitude of change did not differ significantly between the Baduanjin and brisk walking groups (*F* = 0.03, *p* = 0.856). Therefore, no evidence of differential responsiveness between the two exercise modalities was observed.

The responsiveness of the 6MWT was assessed using the standardized response mean (SRM), which was 1.08 (95% CI = 0.84 to 1.31), indicating large responsiveness and strong sensitivity to change following the intervention period. The effect size (*d_p*) based on pre- to post-test change was 0.32 (95% CI = 0.24 to 0.42), representing a small-to-moderate improvement in walking performance. Based on the one-directional improvement criterion, 17 of 70 participants (24.29%) showed an increase in 6MWT distance exceeding the MDC_95_ of 37.19 m and were classified as responders. These included 8 of 34 participants (23.53%) in the Baduanjin group and 9 of 36 participants (25.00%) in the brisk walking group.

## 4. Discussion

To the best of our knowledge, this is the first retrospective study to examine the construct validity and responsiveness of the 6MWT in individuals with schizophrenia receiving exercise interventions. The 6MWT demonstrated excellent long-term stability, acceptable measurement error, moderate construct validity in relation to functional mobility and lower-limb strength, and strong responsiveness to rehabilitation. These findings suggest that the 6MWT is a psychometrically robust tool for monitoring changes in functional exercise capacity among individuals with schizophrenia and that a proportion of patients are likely to show improvements in the 6MWT that exceed measurement error following a 12-week exercise intervention program.

### 4.1. Long-Term Stability and Measurement Error

Previous schizophrenia-specific studies reported ICC values of 0.96 and 0.94 [12,14]; however, these estimates were based on short-interval test–retest reliability and are therefore not directly comparable with the present ICC, which was derived from baseline and post-intervention assessments separated by 12 weeks. Accordingly, the high ICC observed in the present study should be interpreted as supporting the long-term stability of 6MWT scores across the 12-week assessment interval rather than conventional short-interval test–retest reliability. The magnitude of the ICC may also be influenced by relatively homogeneous intervention-related changes and may therefore overestimate stability under more heterogeneous response patterns. Future studies should include two pre-intervention measurements separated by a short, clinically stable interval to allow conventional test–retest reliability and long-term stability to be evaluated within the same cohort.

The MDC_95_ in the present study was 37.19 m, which was lower than the previously reported range of 50–56 m [14]. Differences in sample characteristics, measurement variability, retest intervals, and ICC estimation may have contributed to this difference. Clinically, an increase of at least 37.19 m may be interpreted as individual change beyond measurement error and may assist clinicians in monitoring progress during psychiatric rehabilitation. However, this threshold should not be interpreted as a cutoff of minimal clinically important difference.

### 4.2. Construct Validity

The validity coefficients with the TUG were moderate and inverse, while those with the 30-s Chair Stand Test were moderate and positive, showing both directional and mechanistic consistency. Longer walking distances on the 6MWT reflect greater aerobic capacity and gait efficiency, which are expected to correspond with faster TUG performance and a higher number of sit-to-stand repetitions. Research on balance and functional mobility in schizophrenia has demonstrated that the TUG shows strong reliability and concurrent validity and predicts fall risk more accurately than several static balance measures [20]. The findings support the construct validity of the 6MWT as a clinical measure of lower-limb power, transfer ability, and gait adaptation in schizophrenia. The interpretation of the TUG-cognitive as a validity indicator is nevertheless limited because the accuracy of the concurrent counting task was not recorded. A participant may achieve a shorter completion time by prioritizing gait speed at the expense of counting accuracy; therefore, a shorter TUG-cognitive time may not necessarily represent better overall dual-task performance. This limitation may affect the interpretation of the observed association between the 6MWT and TUG-cognitive performance. Future studies using dual-task measures as validity indicators should record both completion time and cognitive accuracy, such as the number of counting errors, to evaluate possible speed–accuracy trade-offs.

Beyond performance anchors, previous evidence for physiological criterion validity is limited. Gomes and colleagues reported a correlation coefficient of 0.67 between 6MWT distance and peak oxygen uptake in a small sample of men with schizophrenia [12]; therefore, the generalizability of this finding to women remains uncertain. Future studies should examine this association in mixed-sex samples. The disease-specific reference values provided further enhance interpretability by allowing age- and sex-adjusted benchmarking of distance in the 6MWT [13]. Moreover, an earlier review of the 6MWT in schizophrenia by Bernard and colleagues found limited evidence for validity of the instrument against clinically practical anchors [15]. The present findings help address this gap by demonstrating medium-to-large correlations with the TUG and the 30CST in a single cohort.

### 4.3. Responsiveness

The large SRM and the small-to-moderate Cohen’s *d* reflect differences in their standardization terms rather than conflicting findings. The SRM is a group-level responsiveness index derived from individual within-subject change scores and standardizes the mean change by the standard deviation of those change scores. Thus, the SRM of 1.08 indicates that the average improvement was large relative to the variability of change across participants. In contrast, Cohen’s *d* standardizes the mean pre-post difference by the pooled pre- and post-intervention standard deviation and is therefore more strongly influenced by between-participant variability in 6MWT performance. Accordingly, the *d* of 0.32 indicates that the mean change was small to moderate relative to the overall variability of walking distance in the sample. This pattern may reflect relatively consistent changes across participants despite substantial between-participant heterogeneity in walking capacity. In the present study, the SRM was considered the more direct indicator of internal group-level responsiveness, whereas Cohen’s *d* was interpreted as a complementary standardized effect size. At the individual level, 17 of 70 participants (24.29%) exceeded the MDC_95_ threshold, indicating the change may not be attributed to measurement error. However, neither the SRM, Cohen’s *d*, nor the MDC_95_ alone may establish clinically important change; study of such change would require validation against an external patient-centered or clinical anchor.

The large SRM observed in the present study indicates that the 6MWT is sensitive to change over a 12-week rehabilitation period. Responsiveness should be interpreted in relation to the construct and context being measured [25]. More specifically, responsiveness is a measurement property reflecting an instrument’s ability to detect longitudinal change in the construct being measured and is conceptually distinct from the estimation of causal treatment efficacy. Previous psychometric and clinimetric studies have similarly evaluated responsiveness or the ability to detect change using longitudinal score changes, standardized response means, effect sizes, or associations between change scores [28,29,30]. Accordingly, the SRM of 1.08 in the present study should be interpreted as within-intervention responsiveness of the 6MWT among individuals receiving structured exercise rather than as evidence that the observed change was caused exclusively by exercise. For comparison, Pepin et al. reported an SRM of 0.42 for the 6MWT following bronchodilation in individuals with COPD [31], whereas Demers et al. found that the 6MWT was not responsive to pharmacological treatment in patients with heart failure based on effect-size and SRM analyses [32]. These findings suggest that the responsiveness of the 6MWT may vary across clinical populations and intervention contexts, and direct comparisons of SRM values should therefore be interpreted cautiously. The large SRM and the relatively modest responder proportion are not contradictory because they reflect different levels of change. The SRM summarizes the magnitude of change at the group level, whereas the MDC_95_ identifies individual changes that exceed measurement error. The MDC_95_ is derived from the SEM and therefore represents a conservative threshold for detecting individual change beyond measurement error. Thus, participants may contribute to a large group-level response even when their individual improvements do not exceed the conservative MDC_95_ threshold. Because a non-exercise comparison group was not included in the present psychometric analytic cohort, exercise-specific effects could not be separated from learning effects, placebo effects, natural variation, or other nonspecific influences. Future trials should prespecify responder definitions using both MDC and patient-centered anchors and report risk differences and numbers needed to treat relative to controls.

### 4.4. Clinical and Practical Implications

Findings of this present study have implications for research and practice in schizophrenia rehabilitation. First, the combination of excellent stability, moderate validity against clinically feasible anchors, and strong responsiveness supports the use of the 6MWT as a primary measure of functional capacity in psychiatric rehabilitation programs incorporating exercise modalities. Second, approximately one-quarter of participants exceeded the relatively low MDC_95_ threshold and could therefore be confidently classified as responders following time-limited interventions. Moreover, combining changes in distance of the 6MWT with improvements in TUG and 30CST performance may provide a multidimensional assessment of functional gains related to transfers and complex mobility. Third, schizophrenia specific reference values allow for the expression of change as percent predicted or standardized scores, facilitating shared clinical decision-making and individualized goal setting. Clinicians and researchers may use the 6MWT to assess, evaluate, and monitor functional exercise capacity in individuals with schizophrenia. For example, after patients with schizophrenia complete a 3-month exercise intervention, an increase in 6MWT distance exceeding 37.19 m may be interpreted as a real change beyond measurement error. Although such gains may support goals related to walking endurance and community mobility, the MDC_95_ is not equivalent to a minimal clinically important difference, and its relationship with patient-centered recovery outcomes remains to be established. Measures such as the Life-Space Assessment and the Community Balance and Mobility Scale may help determine whether changes in 6MWT performance may translate into broader mobility in everyday and community environments. These measures may complement the 6MWT by extending the assessment from functional exercise capacity to life-space mobility, dynamic balance, and community-based mobility [33,34].

### 4.5. Limitations and Future Directions

Several limitations should be noted. (1) This secondary analysis was derived from studies conducted in a rehabilitation setting, which may limit the generalizability of the findings to acutely ill or highly sedentary individuals and to other clinical settings. (2) The requirement for independent ambulation of at least 50 m may have excluded individuals with more severe mobility impairment, thereby reducing potential floor effects and limiting the generalizability of the findings to patients with poorer functional capacity. (3) Although the 25-m corridor was applied consistently across assessments, it was shorter than the 30-m walkway recommended by the American Thoracic Society. The additional turns required in a shorter corridor may have reduced walking distance and should be considered when comparing the present findings with studies using a 30-m walkway. Corridor length may also have influenced the SEM and MDC_95_. Future studies should directly examine the effects of corridor length on 6MWT performance and related psychometric estimates. (4) Cognitive accuracy was not recorded during the dual-task TUG, precluding assessment of potential speed–accuracy trade-offs. (5) Although one of the parent trials included a health education arm, the present secondary psychometric analysis was restricted to participants receiving structured exercise interventions. Consequently, the observed SRM represents within-intervention responsiveness and does not establish an exercise-specific causal effect. Learning effects, placebo effects, natural variation, and other nonspecific influences therefore cannot be excluded. (6) Although participants were clinically stable, medication-related sedation, extrapyramidal symptoms, and psychomotor slowing were not directly assessed and may have influenced walking performance. (7) The TUG and 30CST were used as clinically feasible functional measures rather than physiological gold standards. Although prior research has shown meaningful associations between the 6MWT and VO_2_ peak [12], no cardiorespiratory exercise test was administered in this study.

The ICC reported in this study reflects the long-term stability of 6MWT scores rather than traditional short-interval test–retest reliability. Short-interval test–retest reliability may differ and should be examined using repeated assessments conducted within a brief and clinically stable interval. Future studies with larger samples should compare long-term stability with short-interval test–retest reliability, examine the potential influence of antipsychotic medication exposure on walking performance, and conduct intervention-specific subgroup analyses for Baduanjin and brisk walking. Larger studies should also evaluate anchor-based responsiveness and establish the minimal clinically important difference in the 6MWT by combining external patient-centered or clinical anchors with distribution-based methods, thereby improving interpretation of clinically meaningful change following exercise interventions.

## 5. Conclusions

In summary, the 6MWT demonstrated excellent long-term stability of scores, moderate construct validity in relation to clinically feasible functional measures, and strong group-level responsiveness over a 12-week exercise intervention period in individuals with schizophrenia. The MDC_95_ of 37.19 m may assist clinicians and researchers in identifying individual changes that exceed measurement error; however, this threshold should not be interpreted as a cutoff of minimal clinically important difference. These findings support the use of the 6MWT as a practical outcome measure for monitoring functional exercise capacity in psychiatric rehabilitation. However, interpretation of these findings should consider the absence of a non-exercise control group and the use of a 25-m corridor, which was shorter than the recommended 30-m walkway. Future studies should use patient-centered or clinical anchors to establish the MCID of the 6MWT in individuals with schizophrenia.

## Figures and Tables

**Table 1:** Demographic and clinical characteristics of the study participants.

Characteristics	Data Value (*n* = 70)
	mean ± standard deviation/ frequency (%)
Age (years)	49.21 ± 9.92
Sex (%)	
Male	37 (52.86)
Female	33 (47.14)
BMI (kg/m^2^)	26.05 ± 4.72
Duration of illness (years)	26.35 ± 8.48
Chlorpromazine equivalents (mg/day)	448.41 ± 236.90
CGI-S scores	3.40 ± 0.67
6MWT (meters)	444.93 ± 65.12
TUG (seconds)	7.82 ± 1.71
TUGcognitive (seconds)	14.53 ± 3.85
30CST (number of repetitions)	14.81 ± 3.46

6MWT: Six-Minute Walk Test; 30CST: 30-Second Chair Stand Test; BMI: body mass index; CGI-S: The Clinical Global Impression-Severity scale; TUG: Timed Up-and-Go Test; TUGcognitive: cognitive Timed Up-and-Go dual task.

## Data Availability

The raw data supporting the conclusions of this article will be made available by the authors on request. The ethical reason is that the information may compromise the privacy of the research participants.

## Abbreviations

The following abbreviations are used in this manuscript:

6MWT	Six-Minute Walk Test
30CST	30-Second Chair Stand Test
CGI-S	Clinical Global Impression–Severity Scale
DSM-5	Diagnostic and Statistical Manual of Mental Disorders, Fifth Edition
ICC	Intraclass Correlation Coefficient
MDC	Minimal Detectable Change
SEM	Standard Error of Measurement
SRM	Standardized Response Mean
TUG	Timed Up-and-Go Test
TUGcognitive	Cognitive Timed Up-and-Go Dual Task
